# Association Between Hospital Performance Metrics and Market Share

**DOI:** 10.1001/jamanetworkopen.2021.30353

**Published:** 2021-10-21

**Authors:** August Oddleifson, Xiao Xu, Patrick Liu, Chengan Du, Erica Spatz, Nihar Desai

**Affiliations:** 1 Center for Outcomes Research and Evaluation, Yale University School of Medicine, New Haven, Connecticut; 2 Department of Obstetrics, Gynecology & Reproductive Sciences, Yale University School of Medicine, New Haven, Connecticut; 3 Section of Cardiovascular Medicine, Department of Internal Medicine, Yale University School of Medicine, New Haven, Connecticut

## Abstract

This cross-sectional ecological study examines the association between publicly reported hospital performance scores in Hospital Care Compare and hospital market share.

## Introduction

There is substantial hospital-level variation in health care quality in the United States.^1^ While there are many tools that provide information to patients about hospital quality and performance, the degree of alignment between hospital performance and market share remains unclear.^2^ The Centers for Medicare and Medicaid Services developed the Hospital Compare website, now referred to as Hospital Care Compare, to enable beneficiaries to make more informed decisions about where to seek care.^3^ This study assessed the association between publicly reported hospital performance scores in Hospital Care Compare and hospital market share.

## Methods

This cross-sectional ecological study used hospital-level, risk-adjusted performance measures and patient volume for the following 3 conditions or procedures available in the Centers for Medicare and Medicaid Services’ Hospital Care Compare database: acute myocardial infarction (AMI), coronary artery bypass graft (CABG), and hip and/or knee replacement. They were selected to exemplify nonelective, semielective, and mostly elective conditions or procedures, respectively. Performance measures included 30-day mortality rate and 30-day readmission rate for AMI and CABG and 90-day complication rate for hip and/or knee replacement for the 2019 report year, reflecting performance in July 1, 2015, through June 30, 2018. Each hospital’s condition- or procedure-specific volume was obtained from the 2020 report year, reflecting patient volumes in July 1, 2016, through June 30, 2019.^4^ The performance score period preceded the volume period by 1 year to account for how consumers have access only to past, as opposed to contemporary, quality measurements. Market share was calculated by dividing the hospital’s volume by the sum of the volume of all hospitals in the hospital referral region (HRR). Hospital and HRR characteristics were derived from the Hospital Care Compare database, the Dartmouth Atlas,^5^ the American Community Survey, and the US Decennial Census.^6^ This research did not meet the National Institutes of Health definition of human subject research as specified in 45 CFR part 46, and was therefore exempt from institutional review board approval. We followed the Strengthening the Reporting of Observational Studies in Epidemiology (STROBE) reporting guideline.

Fractional regressions via generalized linear models with binomial family and logit links were used to model hospital market share for each condition or procedure while adjusting for hospital and HRR characteristics. All estimates reflected marginal effects along with their 95% CIs. Variables adjusted for included number of beds (in tercile), HRR concentration, hospital type (acute care vs critical access), hospital ownership, teaching status, region, HRR population size (in tercile), HRR percentage of the non-White population (in tercile), HRR percentage of the population in rural area (in tercile), HRR percentage of the population with less than a high school education (in tercile), and HRR percentage of the population below the federal poverty level (in tercile). The reference level for tercile variables was the first (lowest) tercile. Terciles were calculated by measure. Two-tailed *P* < .05 was considered statistically significant. Analysis was conducted using RStudio, version 1.2 (RStudio PBC). The eMethods in the Supplement describes additional methodologic considerations.

## Results

Of 4930 hospitals examined, 1993 (40.4%) were included for AMI readmission and 2160 (43.8%) for mortality; 962 hospitals (19.5%) were included for CABG readmission, 971 (19.7%) for mortality, and 2635 (53.4%) for hip and/or knee complication. These hospitals have diverse characteristics (Table 1). The eMethods in the Supplement describes exclusion criteria in detail.

**Table 1.** Descriptive Statistics

Characteristics	No. (%)				
	AMI		CABG		Hip and/or knee complication (n = 2635)
	Readmission (n = 1993)	Mortality (n = 2160)	Readmission (n = 962)	Mortality (n = 971)	
Hospital					
Volume and performance, median (IQR)					
Market share, %	7.3 (2.9-18.5)	6.8 (3.0-16.7)	21.2 (9.2-44.7)	20.9 (9.2-44.4)	5.2 (2.0-13.9)
Score, %	15.7 (15.0-16.4)	12.8 (12.1-13.5)	12.7 (11.9-13.6)	3 (2.5-3.5)	2.5 (2.2-2.9)
Volume, No.	167 (77-309)	155 (77-275)	104 (59-171)	107 (60-174)	211 (88-464)
No. of beds, median (IQR)	223 (137-365)	211 (125-352)	338.5 (226-489.2)	338 (226-492.5)	158 (68-300)
HRR concentration					
Unconcentrated	926 (46.5)	1045 (48.4)	197 (20.5)	215 (22.1)	1164 (44.2)
Concentrated	1067 (53.5)	1115 (51.6)	765 (79.5)	756 (77.9)	1471 (55.8)
Type					
Acute care	1983 (99.5)	2138 (99.0)	962 (100.0)	971 (100.0)	2378 (90.2)
Critical access	10 (0.5)	22 (1.0)	NA	NA	257 (9.8)
Ownership					
Government	229 (11.5)	256 (11.9)	104 (10.8)	106 (10.9)	378 (14.3)
Physician and proprietary	388 (19.5)	424 (19.6)	172 (17.9)	176 (18.1)	496 (18.8)
Voluntary nonprofit	1376 (69.0)	1480 (68.5)	686 (71.3)	689 (71.0)	1761 (66.8)
Teaching status					
Any teaching	215 (10.8)	213 (9.9)	177 (18.4)	179 (18.4)	195 (7.4)
No teaching	1778 (89.2)	1947 (90.1)	785 (81.6)	792 (81.6)	2440 (92.6)
Geographic region					
Midwest	467 (23.4)	496 (23.0)	257 (26.7)	258 (26.6)	761 (28.9)
Northeast	350 (17.6)	404 (18.7)	131 (13.6)	131 (13.5)	400 (15.2)
South	778 (39.0)	846 (39.2)	378 (39.3)	385 (39.6)	935 (35.5)
West	398 (20.0)	414 (19.2)	196 (20.4)	197 (20.3)	539 (20.5)
HRR population, median (IQR)					
Population, No.	1 549 195 (717 277-3 074 036)	1 496 679 (713 457.2-3 074 036)	1 398 813 (641 467.8-2 862 681)	1 398 813 (641 557.5-2 894 926)	1 369 622 (684 888-2 862 681)
Non-White, %^a^	24.1 (15.7-33.9)	24.0 (15.5-33.9)	23.7 (15.7-32.4)	24 (15.7-32.4)	20.9 (14.2-31)
Rural, %	18.7 (8.6-32.7)	18.9 (8.8-34.4)	20.5 (9.4-34.4)	20.5 (9.4-34.2)	22.6 (11.7-37.1)
Less than high school, %	11.1 (9.1-14)	11.1 (9.1-14)	11.2 (9.2-14.2)	11.2 (9.2-14.2)	10.6 (8.9-13.3)
Below federal poverty line, %	17.8 (13.7-20.8)	17.8 (13.7-20.8)	18.0 (14.1-21.5)	18.0 (14.1-21.5)	17.5 (13.6-20.4)

Abbreviations: AMI, acute myocardial infarction; CABG, coronary artery bypass graft; HRR, hospital referral region; NA, not available.

^a^ Demographic characteristics were obtained from the American Community Survey 2015-2019 5-year estimate. Race was determined by self-selection from the following categories: White; Black or African American; American Indian and Alaska Native; Asian; Native Hawaiian and Other Pacific Islander. Respondents were allowed to pick more than 1 response.

For AMI readmission, each percentage point increase in a hospital’s risk-adjusted complication rate was associated with a 1.7 percentage point decrease in its market share (95% CI, −3.10 to −0.25 percentage points; standardized coefficient, −0.16; *P* = .02). There was no significant association between a hospital’s performance score and market share for AMI mortality (−0.85 percentage point; 95% CI, −2.07 to 0.38 percentage points; standardized coefficient, −0.09; *P* = .17), CABG readmission (−0.49 percentage point; 95% CI, −2.49 to 1.52 percentage points; standardized coefficient, −0.04; *P* = .63), and CABG mortality (−1.24 percentage points; 95% CI, −4.40 to 1.93 percentage points; standardized coefficient, −0.06; *P* = .44). For hip and/or knee replacement, each percentage point increase in a hospital’s risk-adjusted complication rate was associated with a 4.2 percentage point decrease in its market share (95% CI, −6.56 to −1.88 percentage points; standardized coefficient: −0.25; *P* < .001) (Table 2).

**Table 2.** Fractional Regression Marginal Effects of Coefficient Estimates^a^

Performance score	Acute myocardial infarction				Coronary artery bypass graft				Hip and knee complication	
	Readmission		Mortality		Readmission		Mortality		Marginal effect, % (95% CI)	*P* value
	Marginal effect, % (95% CI)	*P* value	Marginal effect, % (95% CI)	*P* value	Marginal effect, % (95% CI)	*P* value	Marginal effect, % (95% CI)	*P* value		
Adjusted	−1.68 (−3.10 to −0.25)	.02	−0.85 (−2.07 to 0.38)	.17	−0.49 (−2.49 to 1.52)	.63	−1.24 (−4.40 to 1.93)	.44	−4.22 (−6.56 to −1.88)	<.001
Unadjusted	−3.74 (−5.29 to −2.19)	<.001	−0.81 (−2.13 to 0.51)	.23	−1.89 (−4.11 to 0.34)	.10	−0.21 (−3.61 to 3.18)	.90	−5.66 (−8.27 to −3.06)	<.001

^a^ Calculation of the different components is explained in the Methods section.

## Discussion

The findings of this cross-sectional ecological study suggest that better hospital performance score was associated with larger market share for hip and/or knee replacement and AMI procedures but not for CABG. The effect size was greater for the elective procedure (hip and/or knee replacement) than for the nonelective procedure (AMI), suggesting that patients may be more able to choose their facility for elective procedures.

For CABG, consumers may have less ability to choose their facility based on quality reports owing to the fewer number of facilities available with CABG capacity. While the observed association between hospital performance and market share for hip and/or knee replacement and AMI is encouraging, the effect sizes were modest, highlighting a continued need to better align where patients receive care and the quality of care they receive. This may be achieved by improving patients’ awareness of hospital quality ratings and their ability to choose, by increasing the capacity or geographic accessibility of high-performing centers, or by advancing hospital quality through value-based models of care.

Although we cannot draw causal inferences, these data provide useful initial information about the potential role of public reporting in channeling patients to higher-quality clinicians. Other study limitations include hospital-level analysis in lieu of patient-level analysis and the use of a limited set of performance measures for fee-for-service Medicare beneficiaries only. Further research using patient-level data, other performance measures, and younger patient populations would provide additional insights.

## Notes

Supplement. **eMethods.** Supplementary Methods
eReferences
