# Supplementary material for: Association Between Hospital Performance Metrics and Market Share
Source: JAMA Netw Open. 2021 Oct 21;4(10):e2130353. doi: 10.1001/jamanetworkopen.2021.30353 (PMC8531996; doi:10.1001/jamanetworkopen.2021.30353)
Supplement: Supplement. — eMethods. Supplementary Methods eReferences [file jamanetwopen-e2130353-s001.pdf]

Supplemental Online Content

Oddleifson A, Xu X, Liu P, Du C, Spatz E, Desai N. Association between hospital performance metrics and market share. *JAMA Netw Open*. 2021;4(10):e2130353. doi:10.1001/jamanetworkopen.2021.30353

eMethods. Supplementary Methods

eReferences

This supplemental material has been provided by the authors to give readers additional information about their work.

© 2021 Oddleifson et al. *JAMA Network Open.*

eMethods. Supplementary Methods

Additional Data Sources

A ZCTA to zip code crosswalk was obtained from the American Academy of Family Physicians' Uniform Data System Mapper website and was used to link the ACS ZCTA-level data with zip-code-level HRR designations.[1]

Performance Measures

For all five measures of hospital performance, Medicare beneficiaries 65 or older who were enrolled in fee-for-service Medicare for at least 12 months before their admission were included. For the readmission measures, enrollment for 30 days post-discharge was required. Readmissions were measured within 30 days of discharge. For the mortality measures, deaths from any reason were included, both during the hospital admission or after discharge. The CABG measures were limited to patients undergoing isolated CABG without concomitant valve replacement or other major cardiac or vascular procedure. The hip and/or knee replacement complications included any of the following: acute myocardial infarction, pneumonia, or sepsis/septicemia/shock during the index admission or within 7 days of admission; surgical site bleeding, pulmonary embolism, or death during the index admission or within 30 days; or mechanical complications or periprosthetic joint infection/wound infection during the index admission or within 90 days. CMS conducted a risk adjustment for all five measures to account for patients' characteristics including age, past medical history, and other disease or conditions.[2,3] These measures were chosen because they are widely used by researchers and publicly accessible for patients online. This study centered the performance score at the sample mean.

Exclusion Criteria

The original dataset contained 4,930 hospitals for each of the five performance measures. Ninety-six hospitals met type exclusion criteria (Children's or Veteran's Association). Fifty-nine met state exclusion criteria (territories of Puerto Rico, Guam, Virgin Islands, American Samoa, and Northern Mariana Islands). Nine met ownership exclusion criteria (Tribal). For AMI Readmission, 2,689 did not have an available denominator or score (including hospitals with less than 25 cases who were not eligible for public reporting); 2,517 for AMI Mortality; 3,804 for CABG Readmission; 3,795 for CABG Mortality; and 2,115 for Hip and/or Knee Complication. Two hundred and fifty-nine hospitals did not have a value for one or more covariates for each measure.

Covariates

Hospital characteristics were obtained from the Hospital Care Compare database (geographic region, type/critical access designation, ownership) and the Dartmouth Atlas (bed size, teaching status, and HRR) and reflect calendar year 2017.[4] ZIP Code Tabulation Area (ZCTA)-level demographic characteristics were obtained from the American Community Survey 2015-2019 5-year estimate and aggregated to the HRR-level to measure each hospital's local population size, racial composition (percent non-white), education (percent population with less than a high school education), and poverty (percent population below federal poverty level).[5] Rurality (percent population in rural area) was aggregated to the HRR-level from 2010 US Census data. The degree of market concentration was measured for each HRR using the Herfindahl-Hirschman Index (HHI). HHI is calculated by taking the sum of the square of each hospital's market share in a region and multiplying the sum by 10,000. We considered an HHI below 1500 to be unconcentrated and an HHI of 1500 or above to be concentrated.[6] Other continuous measures of HRR characteristics were categorized using terciles to account for potential non-linear relationship of HRR characteristics with market share.

© 2021 Oddleifson et al. *JAMA Network Open.*

References

1. UDS Mapper. Accessed November 11, 2020. https://udsmapper.org/
2. Centers for Medicare & Medicaid Services. Measure Methodology. Accessed March 23, 2021. https://www.cms.gov/Medicare/Quality-Initiatives-Patient-Assessment-Instruments/HospitalQualityInits/Measure-Methodology
3. Centers for Medicare & Medicaid Services. About the data. Accessed March 23, 2021. https://data.cms.gov/provider-data/topics/hospitals/about-data/
4. The Dartmouth Institute for Health Policy & Clinical Practice. Dartmouth Atlas Data: Downloads. Accessed March 9, 2020. https://atlasdata.dartmouth.edu/downloads
5. United States Census Bureau. American Community Survey (ACS). Accessed March 17, 2020. https://www.census.gov/programs-surveys/acs
6. Cutler DM, Scott Morton F. Hospitals, Market Share, and Consolidation. *JAMA*. 2013;310(18):1964. doi:10.1001/jama.2013.281675

© 2021 Oddleifson et al. *JAMA Network Open.*
